# Exploring the diverse biological significance and roles of fucosylated oligosaccharides

**DOI:** 10.3389/fmolb.2024.1403727

**Published:** 2024-05-28

**Authors:** Burcu Pekdemir, Sercan Karav

**Affiliations:** Department of Molecular Biology and Genetics, Çanakkale Onsekiz Mart University, Çanakkale, Türkiye

**Keywords:** fucose, fucosylation, glycoconjugates, fucosylated oligosaccharides, fucosyltransferases, modifications

## Abstract

Long since, carbohydrates were thought to be used just as an energy source and structural material. However, in recent years, with the emergence of the field of glycobiology and advances in glycomics, much has been learned about the biological role of oligosaccharides, a carbohydrate polymer containing a small number of monosaccharides, in cell–cell interaction, signal transduction, immune response, pathogen adhesion processes, early embryogenesis, and apoptosis. The function of oligosaccharides in these processes is diversified by fucosylation, also known as modification of oligosaccharides. Fucosylation has allowed the identification of more than 100 different oligosaccharide structures that provide functional diversity. ABO blood group and Lewis antigens are among the best known fucosyl-linked oligosaccharides. In addition, the antigens in the ABO system are composed of various sugar molecules, including fucosylated oligosaccharides, and Lewis antigens are structurally similar to ABO antigens but differ in the linkage of sugars. Variation in blood group antigen expression affects the host’s susceptibility to many infections. However, altered expression of ABO and Lewis antigens is related with prognosis in carcinoma types. In addition, many pathogens recognize and bind to human tissues using a protein receptor with high affinity for the fucose molecule in glycoconjugates, such as lectin. Fucosylated oligosaccharides also play vital roles during fertilization and early embryogenesis. Learning and memory-related processes such as neurite growth, neurite migration, and synapse formation seen during the development of the brain, which is among the first organs to develop in embryogenesis, are regulated by fucosylated oligosaccharides. In conclusion, this review mentions the vital roles of fucosylated oligosaccharides in biology, drawing attention to their importance in the development of chemical tools to be used in function analysis and the investigation of various therapeutic targets.

## 1 Introduction

Long since, it was believed that carbohydrates served just as an energy source and structural material and had no biological significance. The biological roles of oligosaccharides, a carbohydrate polymer containing a small number of monosaccharides, have been overlooked in many aspects in the past when compared to the roles of proteins and nucleic acids. In recent years, much has been learned about the biological role of oligosaccharides in cell–cell interaction, signal transduction immune response, pathogen adhesion processes, and apoptosis, and a new field named glycobiology has emerged (Weymouth-Wilson, 1997; Duman et al., 2021). Addition of monosaccharides is catalyzed by specific glycosyltransferases to form oligosaccharides and then these oligosaccharides are bound to proteins and lipids by glycosylation, forming glycoconjugates, the most abundant and structurally diverse class (Miyoshi et al., 2012; Bunyatratchata et al., 2023). The functions of the oligosaccharide units of glycoconjugate classes are difficult to predict. Furthermore, the same oligosaccharide sequence may have different roles at different times in its life cycle or in different parts of an organism. However, by further modifications of common terminal sequences, more specific and crucial biological roles of oligosaccharides or unusual oligosaccharide sequences are diversified (Varki, 1993; Kaplan et al., 2022).

Fucosylation is one of the most considerable types among approximately 10 types of oligosaccharide modifications that play a crucial role in biological processes (Table 1); (Miyoshi et al., 2012). During the fucosylation process, the fucose (Fuc) molecule is bound to oligosaccharides with glycosidic bonds by different fucosyltransferases (FUT). This process has allowed the identification of more than 100 different oligosaccharide structures, providing much of the functional diversity necessary for the development, differentiation, and interactions of complex organisms with other organisms in the environment (Varki, 1993).

**TABLE 1:** Roles and effects of fucosylated oligosaccharides.

Role/Effect	Example	Reference
ABO and Lewis blood group	Contain fucosylated oligosaccharides and differences in expression affect the host’s susceptibility to many infections	Matzhold et al. (2021), Skurska et al. (2022)
Cancer and biomarker	High amounts of fucosyl-linked oligosaccharides in the serum of cancer patients are frequently associated with prognosis	Zhang et al. (2006), Miyoshi et al. (2012), Zhang et al. (2014)
Apoptosis	Changes observed in the oligosaccharide structures of cell surface molecules and fucose residues increase after apoptosis	Russell et al. (1998), Rapoport and Le Pendu (1999), Staudacher et al. (1999)
Host–pathogen interaction and prebiotic effect	Pathogens recognize and bind to human tissues using a receptor with high specificity against fucose on the cell surface or homologs of these structures prevent pathogen attachment	Imberty and Varrot (2008)
Sperm–egg interaction and early embryogenesis	The binding sites on the sperm have a high affinity for the fucosylated oligosaccharides found on the egg. During early embryogenesis, the chains of these oligosaccharides undergo a series of modifications	Dravland and Mortimer (1988), Boldt et al. (1989), Liu et al. (1999), Song et al. (2007)
Neurobiological functions	Fucosylated oligosaccharides regulate nervous system function and development such as neurite outgrowth, neurite migration, and synapse formation	Artavanis-Tsakonas et al. (1979), Okajima and Irvine (2002), Haines and Irvine (2003), Murrey and Hsieh-Wilson (2008)

The connection type and number of Fuc residues show variable characteristics. For instance, in *N-*glycans (oligosaccharides), which are covalently linked to the asparagine (Asn) residue of the protein by an *N*-glycosidic bond, Fuc is attached to galactose (Gal) in α-1,2 or to *N*-acetylglucosamine (GlcNAc) residues in α-1,3, α-1,4, or α-1,6 (Staudacher et al., 1999; Skurska et al., 2022).

**FIGURE 1:**
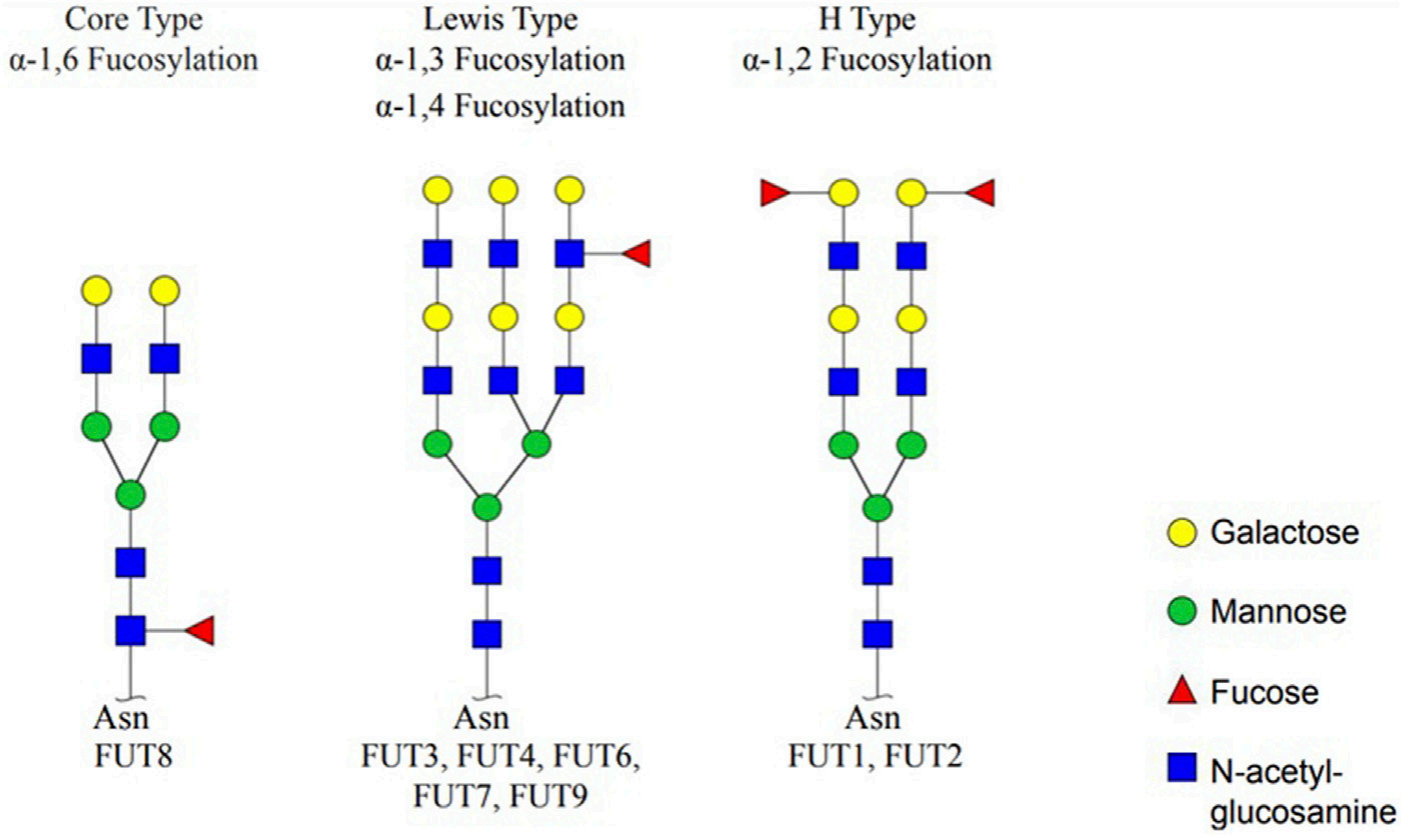
Types of fucosylation and fucosyltransferases. Core fucosylation (α-1,6 fucose), Lewis type (α-1,3/α-1,4 fucose), and H type (α-1,2 fucose) are three fucosylation types. Specific fucosyltransferases; FUT1, 2, 3, 4, 5, 6, 7, 8, and 9 catalyze fucosylation (created by GlycoWorkBench) (Fujita et al., 2021).

There are three types of fucosylation. Fucose is attached predominantly to the first (Asn-linked) GlcNAc residue with α-1,6 and is called α-1,6 fucose (core-type) fucosylation. The other two fucosylation types are α-1,2 fucose (H-type) and α-1,3/α-1,4 fucose (Lewis-type) fucosylation. The fucosylation process is catalyzed by 11 different FUTs, and they are specific for fucosylation types. The H type is catalyzed by FUT1 and FUT2; the Lewis type is catalyzed by FUT3, 4, 5, 6, 7, and 9; and the core type is catalyzed by FUT8. The activity of FUT10 and FUT11 has not been reported (Figure 1) (Fujita et al., 2021).

## 2 Role of fucosylated oligosaccharides in the ABO and Lewis blood group

Fucosyl-linked oligosaccharides have many important roles in biological processes. ABO blood group and Lewis antigens are among the best known fucosyl-linked oligosaccharides. In addition, the antigens in the ABO system are composed of various sugar molecules, including fucosylated oligosaccharides, and the Lewis antigens are structurally similar to the ABO antigens, but differ in the linkage of sugars (Marionneau et al., 2001; Skurska et al., 2022). Lewis antigens are biosynthesized by FUT3. FUT1 and FUT2 are responsible for ABO(H) antigen synthesis, and H antigen is a precursor to create A and/or B antigens (Matzhold et al., 2021; Brockhausen et al., 2022). In hematopoietic tissues, the H gene encodes the FUT1, while the Se secretory gene found in epithelial and mucus secreting cells encodes FUT2 (Comprehensive Glycoscience, 2021). In many tissues, Lewis and ABO antigens are widely expressed, such as in respiratory and gastric mucosa, kidney, endothelium, and heart. Differences in the expression of blood type antigen may affect the host’s susceptibility to many infections (Matzhold et al., 2021). Although enzyme variants and deficiencies result in some well-defined disease, some of the most known enzyme deficiencies may be the reason that formed the basis of blood types in humans. For example, blood type O is caused by enzyme deficiencies and provides health advantages and disadvantages (Comprehensive Glycoscience, 2021). Individuals with blood group O constitute at least 30% of the world’s population. A person with blood type O is thought to be deficient in both A and B glycosyltransferases. ABO blood group antibodies are inherent and are predominantly of the IgM class. In the ABO system, which is known for containing natural antibodies in the bloodstream, the corresponding antibodies may not be detectable in the blood serum. Due to the developing immune systems in newborns, the relatively weak expression of A and B antigens leads to a challenge in accurately identifying naturally produced IgM antibodies against A and B antigens. Consequently, inaccuracies in the use of blood for transfusion and treatment in newborns can result in immune sensitization and post-transfusion complications. According to one hypothesis, anti-ABO IgM is typically absent in newborns but appears within the first year of life. Group-specific antibodies are synthesized in embryos aged 2 to 3 months, thought to be a result of bacterial immunization associated with the influence of intestinal microflora. These antibodies can be produced against food and environmental antigens such as bacterial, viral, or plant antigens that structurally resemble A and B antigens (Gabaidze et al., 2023). These antibodies provide protection to certain infections (e.g., ABO(H) incompatible viruses) and may also interfere with tissue and organ transplantation. Compared to blood type O, blood type A has been shown to protect against severe cholera, while blood type O has been shown to protect against severe *Plasmodium falciparum* parasite infection (Soejima and Koda, 2023). Individuals with the rare Bombay phenotype are characterized by the absence or very weak activity of the α-1,2 fucosyltransferase enzyme encoded by FUT1 and FUT2.

It is clinically important that these individuals with a lack of or weak expression of ABO(H) antigens on the surface of red blood cells can receive autogenous blood or blood transfusion from individuals with the same phenotype only due to the anti-H antibody (Karav et al., 2017). Additionally, the compatibility of fucosylated oligosaccharides is important to prevent organ rejection in organ transplantation (Soejima and Koda, 2023). Differences in blood group antigen expression cause significant advantages and disadvantages in terms of health.

One of the characteristic roles of fucose is regulation of selectin-dependent leukocyte adhesion (Li et al., 2018). Selectins are carbohydrate-binding adhesion molecules necessary for leukocyte trafficking to secondary lymphoid organs and infection sites. Selectin-dependent cell adhesion requires specific positioning and linkage of both sialic acid and fucose residues (Sperandio, 2006; Lühn and Wild, 2012). There are three types of selectins: E-, L-, and P-selectin. L-selectin, the first described member of the selectin family identified in 1982, is expressed on most lymphocytes. E-selectin is rapidly induced by inflammatory cytokines in endothelial cells, while P-selectin is present on the surface of activated endothelial cells and platelets. Selectins bind to sialyl-Lewis X on glycoproteins and glycolipids, including E-selectin ligand-1 (ESL-1), P-selectin glycoprotein ligand-1 (PSGL-1), mucosal vascular addressin cell adhesion molecule-1 (MAdCAM-1), glycosylation-dependent cell adhesion molecule-1 (GlyCAM-1), and CD34 on the endothelium (Li et al., 2018). In this context, fucosylated oligosaccharides that determine blood group antigens play important roles in blood transfusion compatibility, cellular adhesion, and immune responses.

## 3 Role of fucosylated oligosaccharides in cancer

Elucidation of the role of fucosylated oligosaccharides in pathological processes has led to the identification of various types of biomarkers associated with specific types of cancer (Fujita et al., 2021). Changed ABO and Lewis antigen expressions have been seen in all carcinoma types and are frequently associated with prognosis (Zhang et al., 2006; Zhang et al., 2014). Most target glycoproteins that undergo fucosylation are cell surface secretory proteins or membrane proteins. In the liver of cancer patients, fucosylation levels are changed or cellular fucosylation of cancer tissues is increased. Therefore, the sera of cancer patients have shown increased fucosylated oligosaccharides. Normal colon and liver have relatively low fucosylation levels, but during carcinogenesis, the fucosylation level increases (Miyoshi et al., 2012).

In breast cancer cell lines, overexpression of FUT1 or FUT2 has been shown to promote migration, invasion, and metastasis of tumor cells (Fujita et al., 2021). It has also been reported that FUT1 acts as a promoter of cancer progression in oral, hepatocellular, and ovarian cancer by increasing α-1,2-fucose on the cell surface (Lai et al., 2019).

Sialyl Lewis A and X (sLeA and sLeX) are the sialylated forms of Lewis antigens A and X. Both are fucosylated tetrasaccharides, but the position of the fucose linkages differs. The presence of sLeA and sLeX is related with malignancy in various cancer types, and these antigens have been shown to be recognized by selectins, allowing cancer cells to adhere to the vascular endothelium. Therefore, the expression of these antigens, which normally interact with pathogens, in circulating cancer cells facilitates the metastatic process (Miyoshi et al., 2012). The FUT3 gene is also named as the Lewis gene, and high expression of the tetrasaccharide sialic Lewis X, a product of this gene, has been reported in various malignant tumors such as gastric, pancreatic, breast, and ovarian cancer (Mare et al., 2013; Bassagañas et al., 2015; do Nascimento et al., 2020; Dallolio et al., 2021). A study has shown that migration, invasion, and proliferation can be inhibited in gastric carcinoma by silencing the FUT3 gene (Cai et al., 2016). Likewise, FUT4 is highly expressed in lung cancer, and knockdown of FUT4 suppresses apoptosis, thereby increasing chemosensitivity to cisplatin. Hence, it has been reported to prevent epithelial–mesenchymal transition (EMT), cell invasion, adhesion, and migration (Lu et al., 2020). The activity of the PI3K/AKT signal transduction pathway, a key regulator of normal cellular processes including cell growth, metabolism, proliferation, motility, and apoptosis, was found to be modulated by FUT5 and FUT6 expression in colorectal cancer cells (Moafian et al., 2021). FUT7 has been shown to be highly expressed in follicular thyroid carcinoma and is related to poor prognosis (Qin et al., 2020). Additionally, it has been shown in another study that FUT7 expression in urothelial carcinoma is associated with tumor-infiltrating lymphocytes and causes tumor proliferation, invasion, and migration (Liu et al., 2021). Core fucosylation (α-1,6 fucosylation) is catalyzed solely by FUT8. Core fucosylation of the transforming growth factor-beta (TGF-β) receptor is mediated by FUT8 and supports TGF-β signaling and epithelial–mesenchymal transition in breast cancer cells (Tu et al., 2017). FUT8 overexpression has been seen in cancer-related fibroblasts in non-small cell lung carcinoma as well as in prostate cancer cells. Although this causes a decrease in the number of extracellular vesicles secreted by cancer cells, it increases the number of proteins related with tumor metastasis and cell motility (Clark et al., 2020). Studies on colon cancer have shown that FUT9 is related with the expression of cancer stem-related genes. The activity of FUT10 and FUT11 has not yet been fully confirmed in cancer cell types, but meta-analysis of microarray data has shown that FUT11 expression is related with clear cell renal cell carcinoma (Zodro et al., 2014; Lv et al., 2023).

Inhibition of fucosylation, which is related with tumor proliferation and invasion, is being tested to treat carcinomas. Fluorinated fucose analogs prevent the proliferation of various types of cancer cells by blocking the activity of FUTs, including FUT8. As a result, fucosylation is a promising target for cancer treatments (Fujita et al., 2021). In addition, in the humoral immune system, immunoglobulin G (IgG) antibodies play a role in antibody-dependent cell-mediated cytotoxicity (ADCC) targeting natural killer cells and eosinophils against pathogen-infected or cancer cells. This mechanism involves binding interactions where the antibody’s variable region attaches to the target cell and the antibody’s constant (Fc) region binds to the effector cell Fc gamma receptor IIIa (FcgRIIIa). The interaction between IgG1 and the FcgRIIIa receptor is dependent on fucose, and therapeutic IgG1 antibodies with reduced fucosylation at asparagine 297 (Asn297) glycans show increased binding to FcgRIIIa. Afucosylated Fc glycans adopt a high-affinity conformation around tyrosine 296 (Tyr296), forming interactions with the glycan and lysine 128 (Lys128) of FcgRIIIa (Li et al., 2018). Various methods are being investigated to produce therapeutic antibodies with reduced fucosylation for use in clinical trials to determine the significant effects of fucosylation inhibitors in cancer immunotherapy.

## 4 Role of fucosylated oligosaccharides in apoptosis

Apoptosis, known as programmed cell death, is a highly conserved and regulated mode of cell death. In apoptosis, which takes place pathologically in conditions such as cancer, autoimmune diseases, infectious, or neurodegenerative diseases, the oligosaccharide structures of cell surface molecules are changed (Rapoport and Le Pendu, 1999; Staudacher et al., 1999). The fucosylation process also plays a role in maintaining a dynamic steady state in cells and tissues, which is supported by the increase in fucose residues during the apoptosis process.

The findings of a study showed that increased amounts of Fuc residues were observed on the cell surface of mouse thymocytes and P815 cells by three different agents after induction of apoptosis (Russell et al., 1998). In the continuation of the apoptosis process, apoptotic bodies are engulfed by phagocytes, and preventing the release of unwanted molecules and suppressing the inflammatory response is also related with changes in fucosylation (Hall et al., 1994). It has been shown that changes in fucosylation play a role in affecting the activity of tumor necrosis factor-related apoptosis-inducing ligand (TRAIL) in colon cancer. On the other hand, increasing the transcript levels of FUT3 and FUT6 fucosyltransferases showed a positive correlation with TRAIL activity (Haltiwanger, 2009). In a study on human HT-29 colon adenocarcinoma cell lines, strongly enhanced expression of Lewis X and slightly enhanced expression of Lewis Y had been seen on the cell surface prior to cell death (Akamatsu et al., 1996).

## 5 Role of fucosylated oligosaccharides in host–pathogen interactions and prebiotic effect

Cell surfaces contain numerous oligosaccharides, mainly in the form of glycoconjugates that play an important role in intracellular communication. In addition to providing binding to other cells, hormones, and humoral effectors, these oligosaccharides also have a role in the binding of many pathogens to specific extracellular receptors (Newburg, 1997). Pathogens recognize and bind to host glycoconjugates in human tissues using sugar-binding proteins called lectins (Imberty and Varrot, 2008). A lectin family characterized in a previous study was shown to be in the β propeller form. Six symmetrically arranged sugar-binding sites have been identified that bind to glycoconjugates on membranes, also shown to have high affinity for fucose (Kostlánová et al., 2005).

The human milk components such as homologs of host cell surface glycoconjugates, multifunctional substances such as fatty acids and lactoferrin, and secretory antibodies inhibit the pathogens (Eker et al., 2024). Because milk oligosaccharides are synthesized by similar glycosyltransferases that synthesize cell surface glycoconjugates, some of the milk oligosaccharides show structural similarity to cell surface oligosaccharides and may serve as analogs or homologs of cell surface receptors. Therefore, milk oligosaccharides may block the binding ability of pathogens by inhibiting their homologous cell surfaces (Karav, 2018; Bolat et al., 2022). Additionally, most of the oligosaccharide fractions of human milk consist of fucosylated structures. The high affinity of lectin, used as a receptor by pathogens, toward fucose can be blocked by fucosylated milk oligosaccharides (Newburg, 1997).

Expression of fucosylated oligosaccharides in maternal milk varies depending on genetic polymorphisms. Potentially harmful bacteria in the baby microbiota have limited access to oligosaccharides that have been further modified by fucosylation. Because they do not have the necessary glycosyl hydrolase enzymes to break down these monosaccharides, this emphasizes the prebiotic effect of fucosylated oligosaccharides in terms of health (Karav et al., 2016). A study has shown that the content of 2-linked fucosyl oligosaccharides in maternal milk is associated with a lower risk of diarrhea in breastfed infants. While this phenomenon has generally been attributed to the antibody content of maternal milk, research has indicated that these oligosaccharides play a significant role in immunity (Newburg et al., 2004). In another study on this subject, it was shown that fucosylated oligosaccharides obtained from maternal milk prevent infection by preventing the attachment of *Escherichia coli*. These studies provide evidence of the clinical importance of the protection of fucosylated oligosaccharides against infant diarrhea (Newburg, 2000; Morrow et al., 2004).

## 6 Role of fucosylated oligosaccharides in sperm–egg interactions and early embryogenesis

All mammalian eggs are surrounded by the zona pellucida, which plays a vital role during oogenesis, fertilization, and early embryogenesis. This structure is a thick extracellular coat consisting of glycoproteins. The interaction of sperm with the zona pellucida constitutes the first step of fertilization, and the attachment of sperm to this coat is regulated by the glycoproteins found in its structure. Zona pellucida glycoproteins are responsible for promoting acrosomal exocytosis of sperm, limiting or preventing the binding of sperm to unfertilized eggs (Wassarman, 2008). Fucosylated oligosaccharides found on the egg surface have been shown to play a role in fertilization in a wide variety of species. Studies have shown that the high affinity of the binding sites on the sperm to fucosylated oligosaccharides during fertilization negatively affects the sperm–egg interaction if these sites are inhibited by fucose residues. In studies on mammals, the fusion of spermatozoa with eggs in hamster, human, mouse, and guinea pig was inhibited by fucose residues (Dravland and Mortimer, 1988; Boldt et al., 1989; Song et al., 2007). In a study with non-mammals, it was shown that the carbohydrate-binding receptors on the sperm surface were inhibited by fucoidin, known as fucose-sulfate-rich carbohydrates, during fertilization of the brown alga *Fucus serratus* (Bolwell et al., 1979). The same results were shown with similar research on other species such as *Ciona intestinalis*, *Arbacia punctulata*, *Strongylocentrotus purpuratus*, *Strongylocentrotus droebachiensis*, and *Lytechinus variegatus* (SeGall and Lennarz, 1979; Pinto et al., 1981).

During early embryogenesis, which occurs after the fertilization stage and is known for cell division and differentiation, the oligosaccharide structures of glycolipids and glycoproteins on the cell surface undergo changes (Liu et al., 1999). When the molecular mechanism responsible for the expression of fucose-containing differentiation antigens in early embryos was examined, Lewis X was detected in embryo blastomeres. Later, when the blastocyst stage is reached, it has been shown that Lewis X decreases and Lewis Y is expressed at a high rate. In addition to the role of Lewis X and Lewis Y antigens in cell adhesion and implantation, research also focuses on their role in epigenetics, especially at the blastocyst stage. These studies suggest that epigenetic modifications, such as changes in DNA methylation and histone modifications, may be affected by Lewis antigens and their associated glycosyltransferase enzymes (Pennington et al., 1985; Zhu et al., 1995; Liu et al., 1999).

## 7 Role of fucosylated oligosaccharides in neurobiological functions

During embryogenesis, the brain is among the first organs to develop. Fucose is highly abundant in neuronal synapses (Murrey and Hsieh-Wilson, 2008). Fucosylated oligosaccharides have a role during development of the nervous system and its function. Fucosylation process, which affects learning and memory-related processes such as neurite outgrowth, neurite migration, and synapse formation, is required for the activity of Notch, a transmembrane receptor protein that controls a wide range of cell fate decisions (Artavanis-Tsakonas et al., 1979; Okajima and Irvine, 2002; Haines and Irvine, 2003). Fucose has been shown to modulate Notch signaling by directly interacting with Notch ligands or by inducing conformational changes in the protein (Haines and Irvine, 2003). Notch pathways direct brain development by regulating the interactions among cells. During this process, cells switch various genes on and off under the effect of signaling pathways. The Notch signaling pathway plays a role in determining embryonal polarity, maintaining neural plasticity, and regulating neuron functions. The conserved epidermal growth factor (EGF)-like repeats in the extracellular domain of Notch are modified by multiple *O*-fucosylation catalyzed by protein *O*-fucosyltransferase 1 (POFUT1). It is assumed that *O*-fucosyltransferase 2 (POFUT2), an enzyme that modifies thrombospondin type I repeats (TSRs), also plays a role in the folding and quality control of proteins containing TSRs. Both enzymes modify only fully folded substrates, indicating their ability to distinguish between folded and unfolded structures (Vasudevan and Haltiwanger, 2014; Li et al., 2018).

In addition to playing a role in the development and regulation of the nervous system, fucosylated oligosaccharides also play a role in providing tissue homeostasis of microglia, known as the protective cells of the brain. Microglia contain many lysosomes and phagocytic vesicles in their cytoplasm. Lysosomes, the site of catabolic processes of macromolecules, are acidic organelles surrounded by a single membrane. Before deglycosylation of complex or hybrid *N*-glycans with hydrolytic enzymes such as glycosidase, fucose residues catalyzed by α-L-fucosidase must be removed. However, in some cases, the deficiency of this enzyme causes microglia to fail to perform their functions. Fucosylated oligosaccharides resist lysosomal *N*-glycan degradation by negatively affecting the activity of glycosyl hydrolases. As a result, a defect in this enzyme causes the substrate to accumulate, leading to lysosomal storage disease. In further processes, lysosomes play a role in both necrosis and apoptosis of cells (Winchester, 2005).

## 8 Concluding remarks and outlook

The importance of oligosaccharides, a carbohydrate polymer containing a small number of monosaccharides, in their biological roles has begun to be further understood with the advances in the developing field of glycomics. Oligosaccharides link to lipids and proteins through the process of glycosylation, forming glycoconjugates, the most abundant and structurally diverse class. In addition, the oligosaccharide structures or common terminal structures in these glycoconjugates provide functional diversity by identifying more than 100 different oligosaccharide structures with further modification processes such as fucosylation. The fucose molecule, which is attached to oligosaccharides with glycosidic bonds by different fucosyltransferases during the fucosylation process, is different from other naturally occurring sugars. Fucose, the only deoxyhexose sugar found in the L-configuration, is a molecular recognition factor for proteins.

ABO blood group and Lewis antigens are among the best known fucosyl-linked oligosaccharides. In addition, the antigens in the ABO system are composed of various sugar molecules, including fucosylated oligosaccharides, and the Lewis antigens are structurally similar to the ABO antigens, but differ in the linkage of sugars.

Differences in the expression of blood group antigens can alter the host’s susceptibility to many infections. Therefore, fucosylated oligosaccharides that determine blood group antigens also affect the compatibility of blood transfusions and immune responses during transfusion. In addition, the compatibility of fucosylated oligosaccharides is also important to prevent organ rejection in organ transplantation. However, the altered expression of ABO and Lewis antigens has been found in all carcinoma types and is associated with prognosis. Therefore, changes in the expression of fucosylated oligosaccharides also provide the identification of various types of biomarkers associated with certain cancer types. Apoptosis, known as programmed cell death, is a highly conserved and regulated mode of cell death. In apoptosis, which takes place pathologically in conditions such as cancer, autoimmune diseases, infectious, or neurodegenerative diseases, the oligosaccharide structures of cell surface molecules are changed. This change affects the ligand activity that induces TNF (tumor necrosis factor)-related apoptosis. Fucosylated oligosaccharides have also been shown to play a role in pathogen–host interaction. Many pathogens bind to human tissues using a protein receptor, such as lectin, that has a high affinity for the fucose molecule in glycoconjugates. Homologs of these glycoconjugates are highly abundant in maternal milk oligosaccharides, and these milk components may act as analogs or homologs of host cell surface receptors for pathogens. Thus, the lectin with high affinity for fucose, used as a receptor by pathogens, can be blocked by fucosylated milk oligosaccharides. As a result of these studies, it is important to understand the glyco-strategies of pathogens and to contribute to studies on developing glyco-based compounds with anti-adhesion properties against pathogens. In addition to further modifying oligosaccharides by fucosylation, it causes the limited access to oligosaccharides by pathogens due to lack of necessary glycosyl hydrolase enzymes to break down these monosaccharides. Cell–cell interaction of fucosylated oligosaccharides also plays a vital role during fertilization and early embryogenesis. The zona pellucida layer, which is found in mammalian eggs and consists of glycoproteins, is responsible for promoting acrosomal exocytosis of sperm and preventing limited binding or binding of sperm to unfertilized eggs. During fertilization, the high affinity of the binding sites on the sperm to fucosylated oligosaccharides is regulated by changes in fucosylation. During early embryogenesis, which occurs after the fertilization stage and is known as cell division and differentiation, a complex series of changes occur in the fucose diversity of glycoconjugates on the cell surface, and this difference in expression, in addition to playing a role in cell adhesion and implantation, also plays a role in epigenetics, especially at the blastocyst stage. During embryogenesis, the brain is among the first organs to develop and the fucose molecule is abundant in neuronal synapses. Therefore, fucosylated oligosaccharides play a major role in regulating nervous system development and function. Fucosylation process affects learning and memory-related processes such as neurite outgrowth, neurite migration, and synapse formation. Fucosylated oligosaccharides affect the pathways of tissue homeostasis in microglia, known as the protective cells of the brain. Microglia contain many lysosomes and phagocytic vesicles in their cytoplasm. Lysosomes, where catabolic processes of macromolecules take place, are acidic organelles surrounded by a single membrane. Fucose residues must be removed before deglycosylation of complex or hybrid *N*-glycans with hydrolytic enzymes such as glycosidases. In some cases, it causes lysosomal storage disease caused by the deficiency of the α-L-fucosidase enzyme, which is involved in defucosylation.

More detailed studies are needed to understand the mechanism of fucosylated oligosaccharides for the treatment of neurodegenerative diseases that are associated with conditions in which both necrosis and apoptosis occur in later stages. In conclusion, considering the importance of fucosylated oligosaccharides in their biological roles, it is necessary to develop chemical tools for their function analysis, investigate various therapeutic targets, and advance our understanding of the biological process affected by glycoconjugates.
